# Preparation and property analysis of antioxidant of carbazole derivatives

**DOI:** 10.1186/s13065-024-01155-w

**Published:** 2024-03-07

**Authors:** Zhi-quan Zhang, Bao-wei Zhu, Jing-yu Zhang, Hong Chen

**Affiliations:** 1 Department of Chemical and Environmental Engineering, Yingkou Institute of Technology, Yingkou, 115014 Liaoning China; 2 Liaoning Key Laboratory of Chemical Additive Synthesis and Separation, Department of Chemical and Environmental Engineering, Yingkou Institute of Technology, Yingkou, 115014 Liaoning China

**Keywords:** Carbazole, Carbazole derivatives, Lubricating oil, Antioxidants

## Abstract

Carbazole derivatives can be used as antioxidants in the lubricating oil industry. The alkylation of carbazole with 2-chloro-2-methylpropane and 2-bromopropane catalyzed by anhydrous aluminum chloride was studied. Initially, 3,6-di-iso-propylcarbazole and 3,6-di-*tert*-butylcarbazole were using dichloromethane and dibromomethane as solvents at room temperature, respectively. The synthesis conditions were optimized. Subsequently, the effects of reaction time, catalyst dosage, and molar ratio of carbazole to alkylating agent were investigated, and orthogonal experiments were performed. The structures of the carbazole derivatives were characterized by Fourier infrared spectroscopy (FT-IR), mass spectrum (MS) and nuclear magnetic resonance spectroscopy (NMR). The thermal stability of the synthesized carbazole derivatives was investigated by differential scanning calorimetry (DSC). The carbazole derivatives were added into the lubricating oil with a mass fraction of 0.8% and the miscibility, stability and oxidation resistance of the mixed system were evaluated by mechanical stirring and a rotary pressure vessel oxidation test (RPVOT). The DSC results showed that there was good thermal stability for the carbazole derivatives. The mechanical stirring method revealed good solubility and stability for the mixture of oil and carbazole derivatives. The RPVOT results showed that isopropyl carbazole derivatives could increase the oxidation induction period of lubricating oil to 1.39 times, and *tert*-butyl carbazole derivatives could increase the oxidation induction period of lubricating oil to 1.91 times. The antioxidant effect of *tert*-butyl carbazole derivatives was better than that of isopropyl carbazole derivatives.

## Introduction

Mineral lubricating oils inevitably come in contact with light, air, high temperatures, and metals. Oxidation reactions occur, and aldehydes, ketones, and acids are generated under the action of the above factors, which further condense into carbonaceous and asphalt; thus, mechanical equipment is blocked, and the service cycle of lubricating oils is greatly shortened [1–5]. Friction in industrial machinery can easily cause mechanical wear and destruction during machine industry development. About 85% of the reasons leading to machine failure are caused by various forms of friction and wear. Lubrication is the most effective measure to reduce friction and wear [6–8]. Lubricating oils play an important role in the long-term and reliable operation of equipment, implementation of intelligent and green manufacturing, industrial foundations, and other major engineering systems. Various additives are often added to lubricating oil [5, 9–14], among which antioxidants are indispensable and must be added to lubricating oils to slow down the rate of oxidation deterioration to prolong the service life of lubricating oil and significantly improve its thermal stability and antioxidant properties. The oxidation of lubricating oil is essentially a hydrocarbon oxidation process, and its mechanism is a free-radical chain reaction. Researchers at home and abroad have studied the antioxidant properties of some additives; however, there are relatively few studies on new carbazole-lubricating oil antioxidants. Carbazole is an aromatic amine with atoms in the same plane; therefore, it has strong electron conjugation and high thermal stability. Carbazole derivatives are promising high-temperature antioxidants [15–18]. In this study, carbazole-derived lubricating antioxidants were prepared to provide experimental evidence for developing and utilizing aromatic amine antioxidants in lubricating oils.

Carbazole, 2-chloro-2-methylpropane and 2-bromopropane were used as reaction materials, and 3,6-di-*tert*-butylcarbazole and 3,6-diisopropyl carbazole were synthesized by introducing isopropyl and *tert*-butyl, respectively. The introduction of alkyl groups improved the lipophilicity of carbazole. The resulting products were quenched and subjected to salt washing, water washing, extraction, removal, filtration, rotary evaporation, and other related chemical processes to obtain the crude product, which was then separated and purified. The structures of the synthesized products were characterized by infrared spectroscopy, mass spectrometry and nuclear magnetic resonance spectroscopy. The thermal stabilities of the carbazole derivatives were determined by thermogravimetric analysis. The antioxidant properties of the carbazole derivatives were determined using the rotary pressure vessel oxidation test (RPVOT) and differential scanning calorimetry (DSC).

## Materials and methods

We purchased chemical-grade carbazole, anhydrous aluminum trichloride (> 99.5%), *tert*-butane chloride (> 99.5%), bromoisopropane (> 99.5%), dibromomethane (> 99.5%), dichloromethane (> 99.5%), ammonia (> 99.5%), an iodine-free refined salt (> 99.5%), sodium hydroxide (> 99.5%), ethyl acetate (> 99.5%), thin-layer chromatography (TLC) silica gel (> 99.5%), and anhydrous magnesium sulfate (> 99.5%) from Shanghai Macklin Chemicals and used without further purification. We obtained potassium hydroxide (spectral grade), mineral oil (medical grade), and all other chemicals, including petroleum ether (99.9%) (reagent grade), from Tianjin Reagent Chemicals and the lubricating oil from Jiangsu Kuorun Chemical Co., Ltd.

We used Nexus470 (Thermo Nicolet Corporation), SHO193C automatic rotary pressure vessel oxidation test instrument (Shandong Shengtai Instrument Co., Ltd.), DSC-Q20 differential scanning calorimeter (Waters Technology (Shanghai) Co., Ltd.), BRUKER AVANCE III600 MHz nuclear magnetic resonance instrument (Bruker Company, Germany) and a 6460 mass spectrometer (Agilent Technology Co., Ltd.).

### Reaction principle

Under the catalysis of anhydrous aluminum chloride, carbazole reacts with 2-chloro-2-methylpropane to form *tert*-butyl-substituted carbazole derivatives through Friedel–Crafts alkylation at room temperature, according to the following reaction:
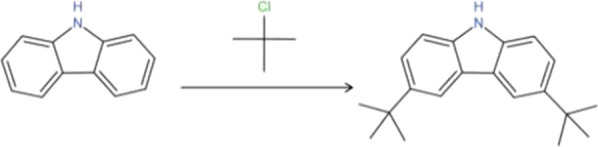


Under the catalysis of anhydrous aluminum chloride, isopropyl-substituted carbazole derivatives were generated by the reaction of carbazole and 2-bromopropane through Friedel–Crafts alkylation at room temperature using the following reaction formula:
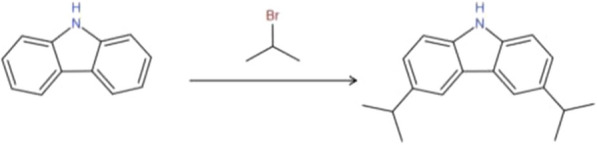


### Preparation of carbazole derivatives

We added certain amounts of carbazole and anhydrous aluminum chloride to a single-port round-bottom flask and used anhydrous dichloromethane as the solvent. We thoroughly mixed the carbazole and catalyst under anhydrous conditions. Carbazole and anhydrous aluminum chloride were completely mixed, dissolved under agitation, and cooled to 0 °C. The 2-chloro-2-methylpropane was dropped into the mixture and stirred quickly at room temperature to make them completely mixed when the temperature of the mixing system reached 0 °C. The reaction ended, and *tert*-butylcarbazole derivatives were obtained after 3 h.

The synthesis of the propyl carbazole derivatives was similar to that of the *tert*-butylcarbazole derivatives, which are not detailed here.

### Separation and purification of carbazole derivatives

After the reaction, we quenched the carbazole derivatives with a high concentration of ammonia washed with distilled water and sodium chloride. We then extracted the quenched, washed, and salt-washed solutions with dichloromethane, collected the organic layer in the lower layer of the separating funnel, and removed the water layer. Anhydrous magnesium sulfate was added to the extracted solution to remove residual water, and the gray-brown solid sample was filtered and dried. We calculated the yields of carbazole derivatives as follows:

$$ {\text{Yield}} = \left( {{\text{experimental}}\;{\text{value}}/{\text{theoretical}}\;{\text{value}}} \right) \times 100\% . $$

### Structure analysis of carbazole derivatives

#### FT-IR analysis

We characterized the structures of the carbazole derivatives by FT-IR spectroscopy and used an infrared (IR) spectrometer with KBr pellets.

#### MS analysis

The samples were dissolved in acetone and analyzed by mass spectrometry. The ion source was ESI ionization. The mode and polarity were set to MS2 scan and positive, respectively.

#### NMR structural characterization

The NMR experiment was performed on a BRUKER AVANCE III 600 MHz NMR spectrometer with an experimental temperature of 298 K, and a solvent of deuterated chloroform. The internal standard was tetramethylsilane (TMS).

### Property analysis of carbazole derivatives

#### Solubility and stability

We added the synthesized carbazole derivative lubricating oil antioxidant to the lubricating oil with a mass fraction of 0.8%. We thoroughly stirred the prepared oil sample using an electric stirrer for 30 min. The mixture was stirred and left to sit before being heated in a water bath. The mixed oil sample and carbazole derivatives were heated to 50–60 °C using a water bath and then stirred for 30 min. We placed the samples into a bottle that was allowed to stand for 48 h to observe the solubility of carbazole derivatives and oil samples and the stability of the mixed system after cooling.

#### Thermal stability

We performed DSC oxidation tests to determine the thermal stabilities of the carbazole derivatives. Both static and dynamic methods are available for differential scanning calorimetry. In this study, we used a dynamic DSC method of differential scanning calorimetry to determine the oxidation stability of the carbazole derivatives. The selected gas atmosphere was nitrogen. We set the programmed temperature at 10 °C/min and the measured temperature range at 0–350 °C. We performed the experimental procedure after the setting.

#### Oxidation resistance

We used the RPVOT method for the antioxidant testing. The synthetic carbazole derivative antioxidant and 50 mL of lubricating oil were added to a quartz cup with a 5 m long copper coil. Next, 5 mL of pure water to the quartz cup to realize a high-humidity environment. The oxygen cylinder was opened, and the automatic oxygenation mode was selected in the RPVOT. Oxygen was filled in the reaction chamber at a pressure of 615 kPa. The reaction temperature was set at 140 °C, and the PRVOT was carried out in an oil bath environment. The quartz cup was at an angle of 30° with respect to the horizontal plane in the reaction chamber, and the rotation speed was 120 r/min. We performed the procedure using the national standard calibration method (SH/T 0193-2008). The experiment was terminated when a specific pressure drop was reached. The oxidation–induction period of the sample was from the beginning to the end of the oxidation [19].

## Results

### Single factor experimental results and analysis

#### Investigation of reaction time

**Fig. 1:**
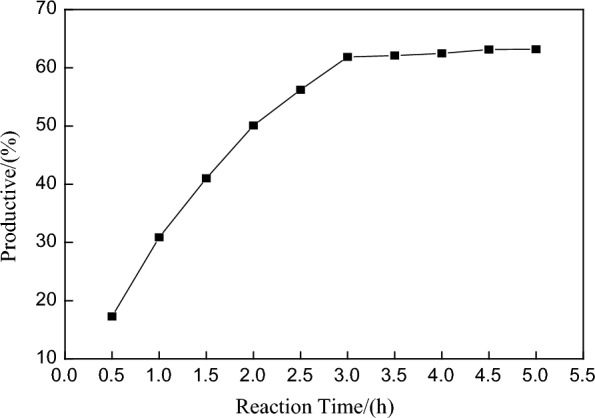
Relationship between reaction time and the yield

The reaction time was changed from 0.5 h to 1 h, 1.5 h, 2 h, 2.5 h, 3 h, 3.5 h, 4 h, 4.5 h, and 5 h for 10 groups of comparative tests to investigate the effect of reaction time on the yield of 3,6-di-*tert*-butylcarbazole. The experimental results are shown in Fig. 1.

Figure 1 shows that the yield of 3,6-di-*tert*-butylcarbazole increased over time. When the reaction time was approximately 3 h, the yield reached a high value, and the increase in the reaction yield was insignificant. Therefore, the reaction time was determined to be about 3 h.

#### Investigation of the ratio of carbazole to anhydrous aluminum chloride

**Fig. 2:**
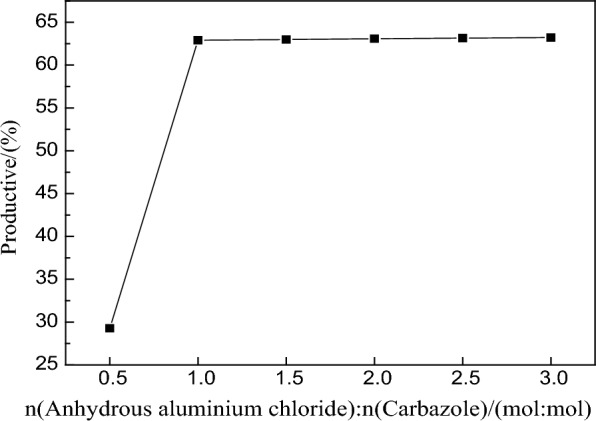
Relationship between molar ratio (n_anhydrous aluminium chloride_:n_Carbazole_) and the yield

We changed the ratio of n (carbazole) to n (anhydrous aluminum chloride) and used the quantitative relationships of 1:0.5, 1:1, 1:1.5, 1:2, 1:2.5, and 1:3 (mol:mol) to conduct six groups of comparative tests. The results are shown in Fig. 2.

As shown in Fig. 2, the yield of 3,6-di-*tert*-butylcarbazole increased with increasing mass of anhydrous aluminum chloride. When the molar ratio of carbazole to anhydrous aluminum chloride was 1:1, the product yield reached a high value, the reaction yield increased slightly, and the increasing trend was very weak. Therefore, the n (carbazole) to n (anhydrous aluminum chloride) was determined to be about1:1.

#### Investigation of the molar ratio of carbazole to 2-chloro-2-methylpropane

**Fig. 3:**
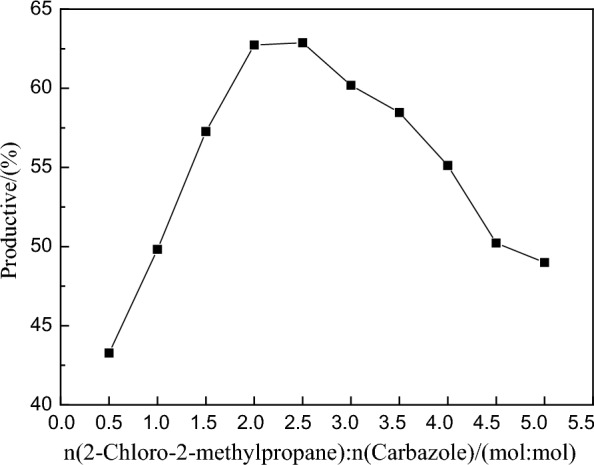
Relationship between molar ratio (n_2-Chloro-2-methylpropane_:n_Carbazole_) and the yield

The ratio of n (carbazole) to n (2-chloro-2-methylpropane) was varied, and quantitative relationships of 1:0.5, 1:1, 1:1.5, 1:2, 1:2.5, 1:3, 1:3.5, 1:4, 1:4.5, and 1:5 (mol:mol) were used for ten groups of comparative tests. The results are shown in Fig. 3.

As shown in Fig. 3, the yield of 3,6-di-*tert*-butylcarbazole first increased and then decreased with an increase in the ratio of carbazole to 2-chloro-2-methylpropane and. When n (carbazole): n (2-chloro-2-methylpropane) was between 1:2 (mol:mol) and 1:2.5 (mol:mol), we achieved the highest yield, and the yield of the reaction decreased gradually with an increase in the content of 2-chloro-2-methylpropane.

### Orthogonal experiment results and analysis

Based on the single-factor experiment, we adopted an orthogonal experiment with three factors and three levels. The factor levels are listed in Table 1, and the experimental results are presented in Tables 2 and 3.

**Table 1:** Experimental conditions

No.	A Time/h	B n_carbazole_:n_2-Chloro-2-methylpropane_	C n_carbazole_:n_anhydrous aluminium chloride_
1	2.5	1:2	1:1
2	3	1:2.5	1:1.5
3	3.5	1:3	1:2

**Table 2:** Orthogonal L_9_ (3^3^) test results

No.	Factors			Yield (%)
	A	B	C	
1	1	1	1	66.24
2	1	2	2	70.77
3	1	3	3	58.93
4	2	1	2	70.52
5	2	2	1	74.72
6	2	3	3	62.47
7	3	1	3	76.21
8	3	2	1	78.77
9	3	3	2	67.13
K_1_	195.94	212.97	219.73	
K_2_	207.71	224.26	208.42	
K_3_	212.11	188.53	197.61	
k_1_	65.31	70.99	73.24	
k_2_	69.24	74.75	69.47	
k_3_	74.04	62.84	65.87	
R	8.73	11.91	7.37	
Order	B > A > C			
Optimal levels	A_3_	B_2_	C_1_	
Optimal conditions	A_3_B_2_C_1_

**Table 3:** Analysis of the orthogonal test

Variation sources	Sum of squares of deviations from the mean	df	Mean square	F
A	0.68	2	0.34	1.37
B	1.18	2	0.59	3.12
C	0.47	2	0.235	1.28

**Fig. 4:**
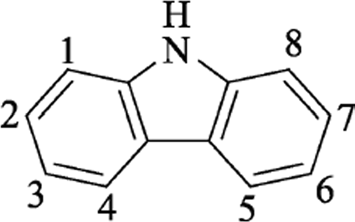
Carbazole molecular

Table 2 shows that the most important factor affecting the yield of carbazole derivatives is the molar ratio of carbazole to 2-chloro-2-methylpropane, the reaction time, and the molar ratio of carbazole and anhydrous aluminum chloride. The variance analysis also indicates that the effect of the molar ratio of carbazole to 2-chloro-2-methylpropane is prominent. The overall trend of the influence of n (carbazole): n (2-chloro-2-methylpropane) on the yield is that the initial rate increases obviously as the amount of *tert*-butyl chloride increases. The yield of 3,6-di-*tert*-butylcarbazole reaches the maximum when the molar ratio of carbazole to 2-chloro-2-methylpropane reaches 1:2.5 (mol:mol). Then, the yield of 3,6-di-*tert*-butylcarbazole decreases with the increase in the content of 2-chloro-2-methylpropane. According to the calculation of static charge distribution on carbazole molecule carbon by Bonesi et al. [12] using the PM3 method, the value of C3 = C6 (Fig. 4) is − 0.126, and the values for C1 = C8, C2 = C7, and C4 = C5 are − 0.107, − 0.082, and − 0.049, respectively. The electron cloud density of C1, C3, C6, and C8 is relatively high and prone to electrophilic substitution reactions. Polysubstituted carbazole may be obtained if the molar ratio of carbazole to 2-chloro-2-methylpropane exceeds 1:2. The optimal molar ratio of carbazole to 2-chloro-2-methylpropane was 1:2 (mol:mol). The optimal reaction time was 3.5 h, and the carbazole to anhydrous aluminum chloride molar ratio was 1:1 (mol:mol). The yield of carbazole derivatives was the highest under the above mentioned reaction conditions.

The optimization process of isopropyl-substituted carbazole was the same as described above.

### Structure characterization and performance analysis

The structure of product was analyzed by FT-IR, MS and NMR. The application performance of the product was tested by DSC and RPVOT methods.

#### FT-IR analysis

**Fig. 5:**
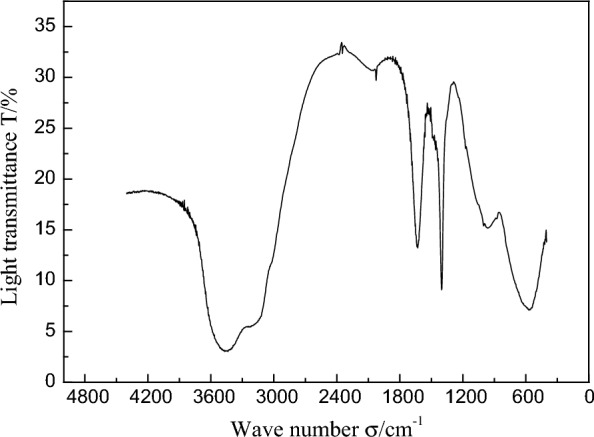
Fourier infrared spectra of 3,6-di-*tert*-butylcarbazole

Figure 5 shows the infrared spectrum of 3,6-di-*tert*-butylcarbazole, taking 3,6-di-*tert*-butylcarbazole as an example. The infrared absorption peaks are at 3450 cm^−1^, 1650 cm^−1^, and 1390 cm^−1^, where 3450 cm^−1^ is the stretching vibration peak of –NH– and 1650 cm^−1^ is the absorption peak of five-membered heterocyclics. 1390 cm^−1^ is the infrared absorption peak of the *tert*-butyl group. We determined the synthesized carbazole derivative to be 3,6-di-*tert*-butylcarbazole according to the results of the infrared spectrum analysis.

#### MS analysis

**Fig. 6:**
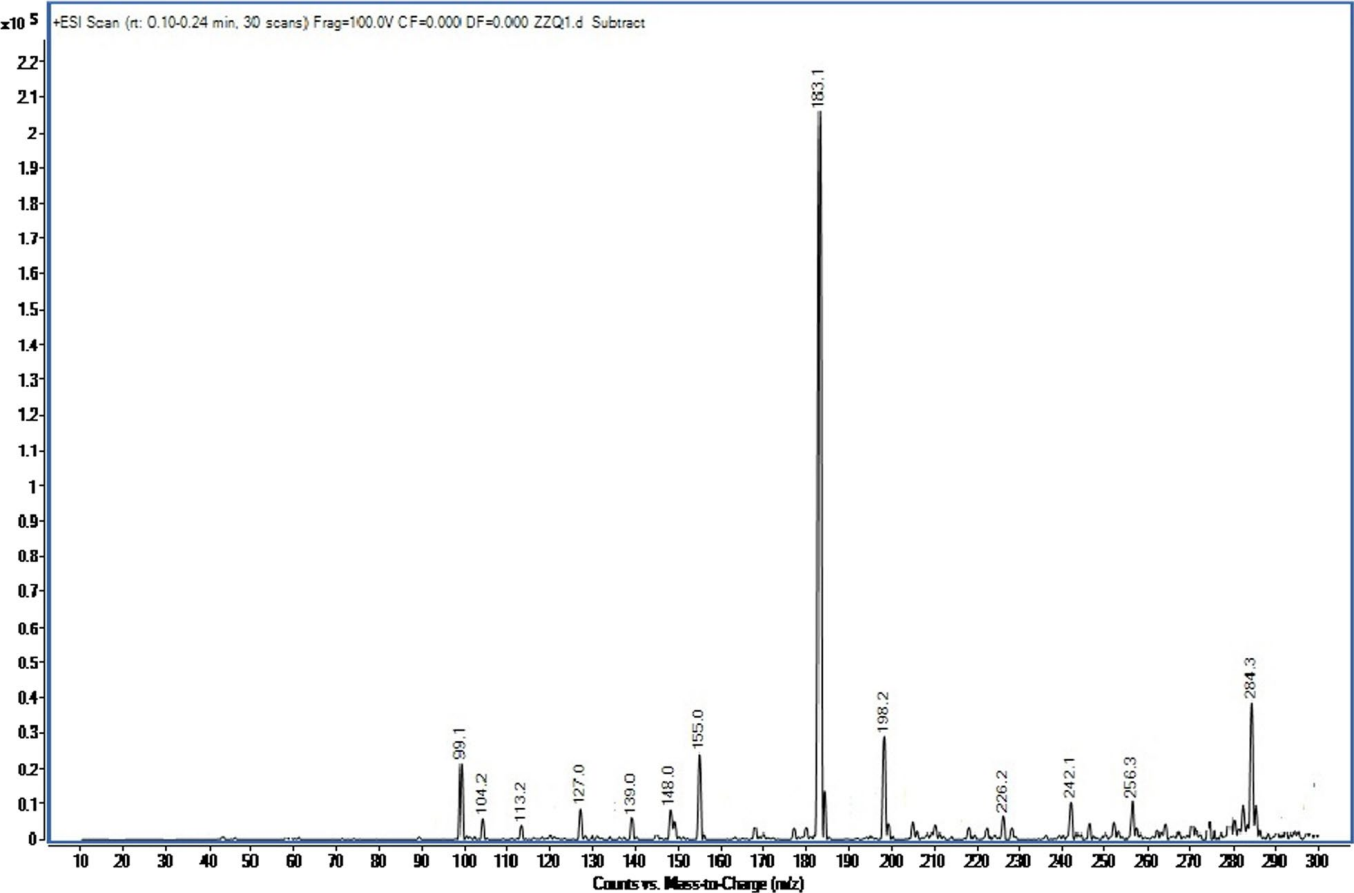
MS spectra of 3,6-di-*tert*-butylcarbazole

Figure 6 shows the mass spectrum of the target product. The maximum mass/nucleus ratio of the unknown molecule is 284. The relative molecular weight of the unknown substance is 284, and the synthesized carbazole derivatives should be 3,6-di-*tert*-butylcarbazole according to the molecular ion peak and several fragment ion peaks with high abundances, such as M/z = 198, M/z = 183, and M/z = 155.

The location of the first fragment ion peak was M/z = 198, and the difference between the fragment ion peak and the relative molecular mass of 284 was M/z = 86, indicating that 3,6-di-*tert*-butylcarbazole was rearranged after the ion source crushed *tert*-butylcarbazole. The position of the second fragment ion peak was M/z = 183, and the difference in the mass/nucleus ratio between the fragment ion peak and the first fragment ion peak was M/z = 15, which is the fragment ion peak generated when –NH– was shot down after 3,6-dimethylcarbazole was bombarded with an ion source.

In the position of the third fragment ion peak was M/z = 155, and the difference in the mass/nucleus ratio between the fragment ion peak and the second was M/z = 28, which was the fragment ion peak generated after the two methyl groups were smashed by ion source bombardment. The last position with high abundance was M/z = 99. The fragment peak formed after the benzene ring was rearranged to stabilize its structure.

#### NMR analysis

The structure of 3,6-di-*tert*-butylcarbazole was characterized by ^1^H-NMR and ^13^C-NMR. The target product ^1^H-NMR (600 MHz, CDCl_3_), δ:8.04 (s, 2 h), 7.82 (s, 1 h), 7.42 (d, J = 8.6 Hz, 2 h), 7.33 (d, J = 8.6 Hz, 2H), 1.45 (s, 18H); ^13^C-NMR (100 MHz, CDCl_3_), δ: 142.33, 138.04, 123.53, 123.35, 116.18, 109.99, 34.71, 32.05. The ^1^H-NMR and ^13^C-NMR spectrum show that two positions on the benzene ring are replaced, and the two positions are located on the two benzene rings and the positions are symmetric.

We determined the synthesized carbazole derivatives to be 3,6-di-*tert*-butylcarbazole based on their infrared spectra, mass spectra, and NMR.

Compared with the relevant literature, the synthesis method of this experiment is simple. The experimental conditions do not need to be carried out under vacuum and oxygen-free, and the product yield is higher.

#### Solubility and stability test

The experimental results showed that carbazole-derived antioxidants dissolved well in lubricating oil and kept the lubricating oil transparent, taking the compatibility and stability of 3,6-di-*tert*-butylcarbazole as an example. After 48 h, the prepared oil samples remained clear, transparent and total acid value is 0.09 mgKOH/g. The lubricating oil sample became turbid when we added carbazole directly to the lubricating oil. We increased the carbazole derivatives’ solubility in lubricating oil by introducing *tert*-butyl groups in the benzene ring of carbazole, and the stability of the synthesized carbazole derivatives and oil was good. The oil samples prepared with carbazole derivatives were still in a clear and transparent state after 30 d of standing, indicating that the introduction of alkyl groups in the benzene ring of carbazole increased the solubility of carbazole in the base oil and the stability of the oil was relatively good. The TG curve of the base oil after antioxidant addition is shown to the right of the TG curve of the base oil without antioxidant addition. Qualitative analysis showed that the base oil's thermal stability improved after adding antioxidants.

The oxygen degradation of lubricating oil leads to changes in its chemical composition, which in turn affects its physical and chemical properties. Based on the cost performance of the additive amount of antioxidants, we selected the additive amount of carbazole derivatives as 0.8%. We investigated the change in kinematic viscosity with oxidation time using an oven-accelerated oxidation experiment. The results showed that the kinematic viscosity of lubricating oil without 3,6-di-*tert*-butylcarbazole antioxidant increased rapidly with oxidation time, and the kinematic viscosity was 13.24 mm^2^/s after oxidation for 24 h and 20.52 mm^2^/s after oxidation for 10 days. After adding antioxidant 3,6-di-*tert*-butylcarbazole 0.8%, we inhibited the increase of kinematic viscosity to a certain extent. The effect was more apparent, and the kinematic viscosity only increased to 15.12 mm^2^/s after 10 days of oxidation. Compared to the base oil, the oxidation process of the oil was significantly slower, and the increase in kinematic viscosity was inhibited.

#### Differential scanning calorimetry experiment result analysis

**Fig. 7:**
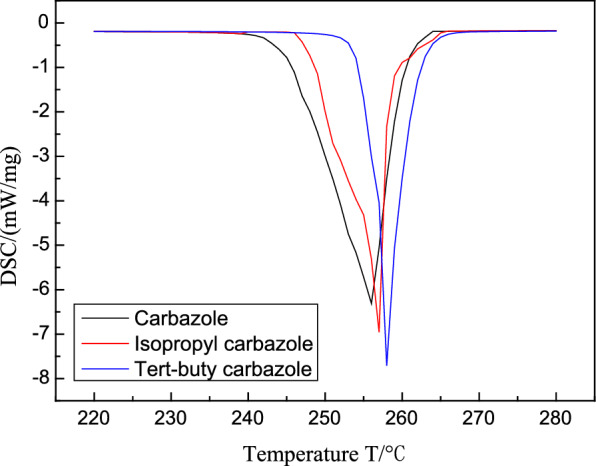
DSC dynamic results of carbazole antioxidants

We obtained DSC curves of the carbazole, isopropyl carbazole, and *tert*-butylcarbazole derivatives using a thermal analyzer after drying and grounding the products. Figure 7 shows that the DSC curves of the carbazole derivatives are all on the right side of that of carbazole, and the curve slopes of the carbazole derivatives are higher than those of carbazole. Therefore, the oxidation stabilities of the two carbazole derivatives improved.

#### RPVOT analysis

**Fig. 8:**
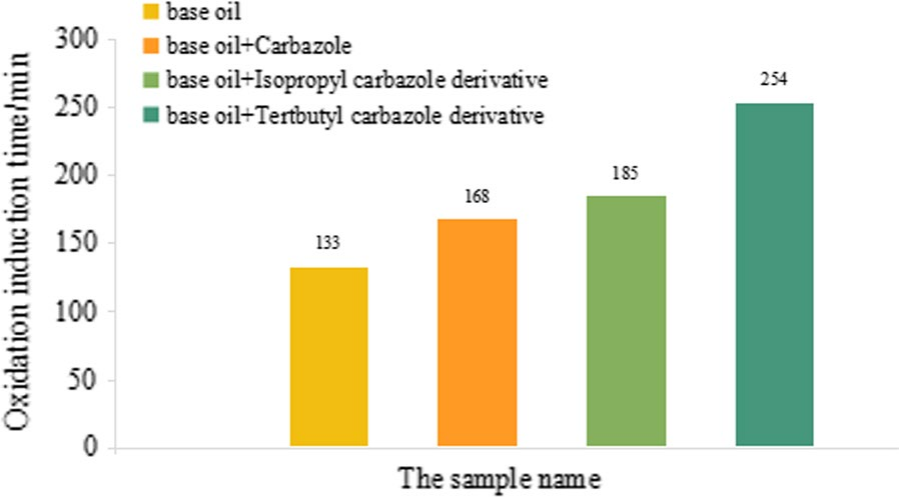
RPVOT analysis results of carbazole derivative antioxidants

Carbazole derivative antioxidants were added to the lubricating oil using the 1.5.3 method to prepare the sample. We determined the oxidation induction period of oil samples by the RPVOT method according to the SH/T 0193-2008 standard, and the results are shown in Fig. 8.

Figure 8 shows that the period of the lubricating oil was 133 min and 168 min after we added carbazole to the lubricating oil, which was approximately 1.26 times of the base oil. The oxidation induction period of the lubricating oil was 185 and 254 min after the isopropyl carbazole and *tert*-butylcarbazole derivatives were added to the lubricating oil, which were approximately 1.39 and 1.91 times of base oil, respectively. Carbazole derivatives can be used as free radical scavengers because of the relatively active H on N. Carbazole can combine with the free radicals generated in the oxidation process of lubricating oil, terminate the growth of free radicals, and cut off the chain reaction. It is also a hydrogen donor, but the free radical formed after replacement of H is very stable under strong conjugation, and it becomes difficult to continue the reaction. Therefore, the *tert*-butyl carbazole derivative exhibited better oxidation resistance as a lubricating oil additive. When *tert*-butyl is added to the benzene ring of carbazole, all the atoms are in the same plane, and the structure of the carbazole derivatives is extremely stable. The results showed that carbazole derivatives, especially *tert*-butylcarbazole derivatives, can prolong lubricating oils’ antioxidant time and service life. Notably, 3,6-di-*tert*-butylcarbazole can be used as a free radical scavenger because of its relatively active H on N, which can combine with free radicals generated in the oxidation process of lubricating oil, terminate the growth of free radicals, and cut off the development of chain reactions. The essence of the process is the hydrogen donor, and the free radical formed after carbazole gives H, which is very stable under strong conjugation, making it difficult to continue the reaction. Therefore, carbazole derivatives as lubricant antioxidants can reduce the oxidation rate of lubricating oil.

## Conclusions

Two carbazole derivatives were prepared via orthogonal experiments. The most influential factor in the synthesis of *tert*-butylcarbazole derivatives was the mass ratio of carbazole to 2-chloro-2-methylpropane, followed by the reaction time and mass ratio of carbazole to anhydrous aluminum chloride, as determined by means and range analysis of the orthogonal experiment. The optimum reaction conditions for the synthesis of 3, 6-di-*tert*-butylcarbazole were as follows: reaction time of 3.5 h, mass ratio of carbazole to 2-chloro-2-methylpropane of 1:2.5 (mol:mol), and mass ratio of carbazole to anhydrous aluminum chloride of 1:1 (mol:mol). The synthesis conditions for 3,6-di-isopropyl carbazole were as follows: reaction time of 3 h, mass ratio of carbazole to 2-bromopropane 1:2.5 (mol:mol), and mass ratio of carbazole to anhydrous aluminum chloride 1:1.5 (mol:mol). The synthesized carbazole derivatives were characterized by IR, MS and NMR. The synthesis method is simple and easy to operate, the experimental conditions do not need to be carried out under vacuum and oxygen-free conditions, and the product yield is higher. Among the prepared carbazole derivatives, the oxidation resistance of *tert*-butylcarbazole derivatives was better than that of isopropyl carbazole derivatives. Within a certain range, the longer the chain of substituents, the more beneficial the antioxidant.

The properties of the carbazole derivatives were determined using mechanical stirring, DSC, and RPVOT. The solubilities of the carbazole derivatives and lubricating oil mixtures were satisfactory. The prepared oil samples were transparent, clear, and stable. The DSC experimental results showed good thermal stability, and the RPVOT performed well as an antioxidant additive in lubricating oil for carbazole derivatives.

## Data Availability

Data will be available on request by the corresponding author.
